# Gonad shielding in pelvic radiography: modern optimised X-ray systems might allow its discontinuation

**DOI:** 10.1186/s13244-019-0828-1

**Published:** 2020-02-07

**Authors:** Cécile R. L. P. N. Jeukens, Gerhard Kütterer, Pierre J. Kicken, Marij J. Frantzen, Jos M. A. van Engelshoven, Joachim E. Wildberger, Gerrit J. Kemerink

**Affiliations:** Department of Radiology and Nuclear Medicine, Maastricht University Medical Center, P. Debijelaan 25, 6229 HX Maastricht, The Netherlands

**Keywords:** Gonad shielding, Pelvic radiography, Gonad radiation dose, Hereditary radiation risk, Optimisation

## Abstract

**Objective:**

As gonad shielding is currently under debate, this study evaluates the practice, from its introduction in about 1905 until today.

**Methods:**

The literature was searched for developments in shielding and insights into the effects of ionising radiation on gonads. Based on own pre-1927 dose reconstructions, reported doses after 1927, a 2015-report from the European Union and recent own measurements, the effects of technological evolution and optimisation on radiation dose and hereditary risk were assessed.

**Results:**

In the 1900s, gonad shielding was first applied to prevent male sterility, but was discontinued when instrumental developments led to reduced radiation doses. In the 1950s, concerns about hereditary risks intensified and gonad shielding was recommended again, becoming routine worldwide. Imaging-chain improvements over time were considerable: in 2018, the absorbed dose was 0.5% of its 1905 value for the testes and 2% for the ovaries, our optimised effective dose a factor five lower than the value corresponding to the current EU diagnostic reference level, and the reduction in detriment-adjusted risk by shielding less than 1 × 10^−6^ for women and 5 × 10^−6^ for men.

**Conclusions:**

Assessment of pelvic doses revealed a large reduction in radiation risks facilitated by technological developments. Optimisation likewise contributed, but unfortunately, its potential was never adequately exploited. Today, using a modern and optimised X-ray system, gonad shielding can be safely discontinued for women. For men, there might be a marginal benefit, but potential negative side-effects may well dominate. Discontinuation of gonad shielding seems therefore justifiable.

## Key points

- Gonad shielding originated around 1905 to prevent male sterility, but was discontinued after doses went down
- In the mid-1950s, gonad shields were reintroduced, now to reduce hereditary risks
- Technological evolution and optimisation lowered gonad doses to 0.5–2% of the 1905 values
- Today, after optimisation, the hereditary risk reduction is marginal at best (< 5 × 10^−6^)
- Considering also negative side-effects, discontinuation of gonad shielding seems justifiable

## Introduction

The benefit of gonad shielding in anteroposterior (AP) pelvic radiography is currently under debate. The ICRP (2013) [1] and IAEA (2018) [2] endorse this practice, whereas others, such as the Dutch guidelines [3], Marsh and Silosky [4] and the AAPM [5], no longer recommend it. Other authors dismiss gonad shielding partly or express their doubts about existing benefits [6–13]. This debate should ideally be decided by a quantitative analysis based on proper knowledge of radiation risks, reduction in hereditary risk by gonad shielding and the increase in risk caused by negative side-effects of shielding. The Dutch guidelines [3] provide steps in these directions, as does the work by Frantzen et al. [7].

This article aims at a missing, more complete assessment of benefits and risks of gonad shielding, from its beginning until now. To be addressed are the following: the histories of gonad shielding and perception of gonadal radiation risk, the evolution of the dose of a pelvic radiograph, imaging chain improvements and the decrease in detriment-adjusted risk by gonad shielding. The historical aspects are presented within ‘Introduction”, the other three in “Methods and materials, Results and Discussion’.

### Perception of gonadal radiation risk: a historical overview

The similarity between X-ray erythema, already observed in 1896 [14], and the erythema caused by ultraviolet radiation applied in the so-called Finsen therapy of skin diseases [15], probably led the way to therapeutic applications of X-rays. As early as 1901, Williams reported about a dozen benign and malignant skin afflictions which were treated with X-rays [16]. Amongst these were eczema of the scrotum, tuberculosis of the testes and pruritis ani [17–20]. Clearly, no barriers were felt at that time to expose the testes to very high radiation doses.

Already in 1896, X-rays had been used for “deep therapy” [21], albeit with limited success. In 1903, Albers-Schönberg studied the effect of X-rays on the testes, finding that male rabbits and guinea pigs could easily be sterilised, even without inducing dermatitis of the skin [22]. In 1905, Halberstaedter similarly found high radiation sensitivity for the ovaries of rabbits [23]. Temporary and permanent sterility of male operators of X-ray systems was reported not long thereafter [20].

Biological effects of radiation at the level of tissues, cells and chromosomes were also studied from the beginning. In 1906, Bardeen wrote an extensive overview of these experiments [24]. In his own studies on toads, he found that irradiated sperm, notwithstanding the apparently normal fertilisation of eggs, resulted in abnormal development. Damage to the chromosomes was the cause. The fact that radiation-induced mutations could also be inherited was proven by Muller in 1926 [25]. Mavor had already shown this in 1921 [26], but he was somehow not given the credits. Muller assumed no threshold in the induction of heritable mutations, a proposition still held today. Soon thereafter, concern for hereditary effects in radiology was expressed in the literature [27–31]. After World War II, the fear for radiation was fuelled by the effects observed in victims of the nuclear bombs on Hiroshima and Nagasaki. Apprehension grew further due to the increasing exposure to radiation, from medical applications, nuclear industry and, at that time, fall-out of nuclear bomb testing.

Even though no radiation-induced genetic effects had been observed, the ICRP worried about the accumulation of genetic mutations, leading in 1956 to the declaration: “Genetic damage assumes greater importance” and “Realising the importance and urgency of the matter….. to recommend in the near future a maximum permissible ‘genetic dose’….” [32]. Soon afterwards, the genetically significant dose (GSD) was introduced as a measure for the annual radiation load of the genome of the whole population. UNSCEAR explained in 1958 “…., a genetically significant dose can be defined as the dose which, if received by every member of the population, would be expected to produce the same total genetic injury to the population as do the actual doses received by the various individuals” [33].

In 1958, the ICRP suggested a genetic dose limit of 5 rem (50 mSv) per generation [32]. The GSD was assessed in numerous studies. In 1969, the ICRP informed “The genetically significant dose from medical diagnostic radiology has been determined for many countries and ranges between 10 and 60 mrad per annum” (0.1–0.6 mGy/year) [34]. As such, over the 30 years usually considered for procreation, the genetic dose was lower than the ICRP limit and also lower than the dose due to natural radiation. The GSD has quietly disappeared from contemporary literature. The reasons are probably the non-alarming values and the smaller than feared hereditary effects. Cancer induction became the dominating concern [35].

Since 1977, the genetic risk is, together with the somatic risk, included in the effective dose equivalent (*H*_E_), later redefined as the effective dose (*E*). An earlier effort to combine genetic and somatic risk in a “Gesamtbelastung” was proposed by Frik in 1960 [36].

The changing insights into the risk of genetic effects are reflected in the decreasing tissue weighting factor for the gonads used in the calculation of the effective dose equivalent or effective dose: 0.25 in ICRP 26 (1977) [37], 0.20 in ICRP 60 (1990) [38] and 0.08 in ICRP 103 (2007) [35].

Table 1 shows some effects of X-rays on gonads (after ICRP 103) [35].

**Table 1:** Estimates of the threshold absorbed dose for tissue effects in adult human gonads [35]

Tissue	Induction of sterility	Total dose in single exposure (Gy)	Total dose in protracted exposure (Gy)	Annual dose rate in protracted exposure (Gy/year)
Testes	Temporary	0.15	–	0.4
	Permanent	3.5–6.0	–	2.0
Ovaries	Permanent	2.5–6.0	6.0	> 0.2

Note: hereditary effects are assumed to be induced with no dose threshold

### Gonad shielding: its introduction, hardware and recommendations

Since Röntgen’s first X-ray experiments, lead (Pb) was the preferred material for shielding. Unfortunately, lead contaminates hands and clothing and it creases after repeated use. In 1903, Holzknecht succeeded in covering lead foil with rubber, eliminating contamination as well as the formation of sharp folds and holes by repeated bending [39].

As early as 1905, Cramer [40] used gonad shielding during therapy on both male and female patients, as did Halberstaedter [23] on females. In 1907, Kienböck recommended shielding of the testes whenever possible, both during diagnostic and therapeutic X-ray exposure [41]. Albers-Schönberg did the same in the 1910, 1913 and 1919 editions of his famous textbook “Die Röntgentechnik” [42]. The advice to shield the testes was absent, however, in the 1941-edition, appearing 20 years after his death with Grashey as editor [43]. Recommendations to shield the ovaries were not found in early literature on diagnostic radiology, as opposed to therapeutic radiology.

In 1954, the ICRP wrote with respect to radiology, referring to both male and female patients, “In all irradiations the gonads should be protected as much as possible by collimation of the beam or by protective screens.” [44].

**Fig. 1:**
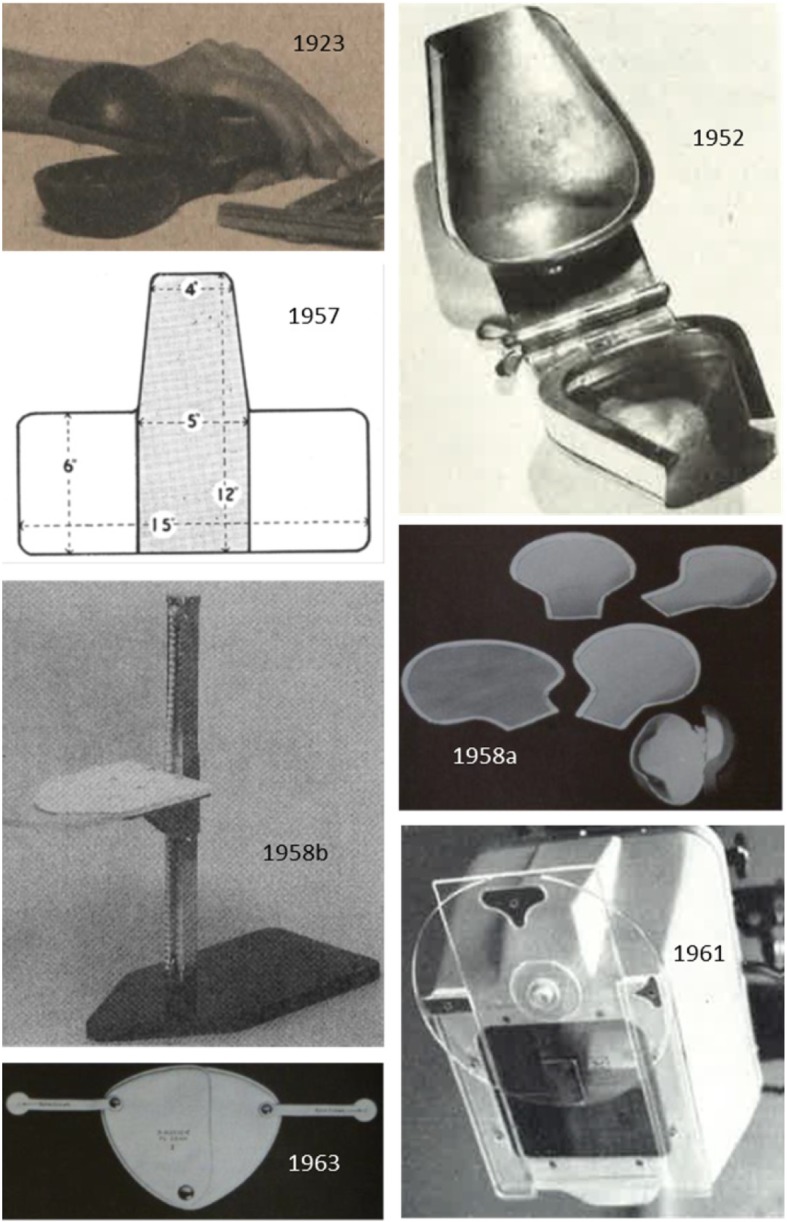
Some early gonad shields with the year they were described in the literature. Testes capsules are seen on the photographs from 1923 [45], 1952 [46] and 1958a (at the right bottom of the image) [47]. A PMMA T-shaped board with 2-mm lead (shaded) for testes shielding is shown on the 1957 drawing [48]. Flexible contact-type shields for females are seen on the 1958a [47] and 1963 [55] images. The devices on the photos from 1958b [52] and 1961 [53] are of the projection type, the first to be positioned somewhere above the patient and the latter was fixed to the X-ray diaphragm

Many different types of gonad shielding were proposed: capsules for the testes [45–47], flat contact-type shields [47–51] and projection-type shields consisting of a shield mounted on a stand [52] or on a PMMA rotatable disk to be mounted on the diaphragm housing [53, 54]. For more early designs and references, see Büchner [55], Stieve [49], Markó [56] and Grigg [57]. Even internal shielding of a foetus and the ovaries by introducing BaSO_4_ into the gastrointestinal tract has been proposed [58]. A selection of shields is shown in Fig. 1 [45–48, 52, 53, 55].

In general, contact shields prevail, with size and shape dependent on age and gender of the patient [47–51].

Endorsed by national and international bodies, gonad shielding became routine. ICRP 34 [59] states “The gonads of individuals with reproductive potential should be protected if they are within the primary beam or within 5 cm of it, and if the shielding does not exclude important diagnostic information or interfere with the study.” Gonad shielding can lower the dose to the testes by about 95% and to the ovaries by about 50% [59]. The protection in females is less effective, mainly due to the large variation in the position of the ovaries, including areas far from the midline lying anterior to pelvic anatomy which must remain visible [6]. In practice, it is difficult to position the X-ray shield correctly, i.e. fully covering the target area but none of the bony pelvic structures: in a meta-analysis, based on 19 studies, the average of correctly positioned shields was found to be only 34% [60].

## Methods and materials

### Radiation dose of an AP pelvic radiograph over time

To appreciate the benefit of gonad shielding in pelvic radiography, knowledge of the dose incurred by the testes and ovaries is required. Therefore, dose information was sought from the start of radiology in 1896 up to 2018. Unfortunately, it turned out that effectively no explicit doses had been published before 1927. Exposure parameters were found in the literature, however, and these could be used for dose reconstruction with an estimated uncertainty of 40–60% (a typical dose reconstruction required about seven parameters, each with its own potential error, which explains the large uncertainty). After 1927, explicit doses were reported and these have been collected. All dose data, reconstructed and retrieved, were presented as “entrance surface air kerma including backscatter” (ESAK). Because of its large size, this study was published separately [61]. Using the ESAK values obtained, it is possible to estimate effective dose and gonad doses by first converting the ESAK values to kerma free in air (KfiA) (by dividing ESAK by the backscatter factor) and then using the KfiA as input in PCXMC [62, 63]. PCXMC is a Monte Carlo programme for computing patient doses in radiology.

Doses were calculated for three landmark times. For the time at which gonad shielding was introduced, three representative results from 1904–1906 were averaged [64–67]. Similarly, for the time gonad shielding was reintroduced in the mid-1950s, the 1958 cases from Janker [68] and Lincoln [69] were assessed. Finally, for recent times, data from one European and two Dutch sources have been used:

- The most common European diagnostic reference level (DRL) for anteroposterior (AP) pelvic radiography, specified by a kerma area product (KAP) of 3.0 Gy cm^2^ [70], for doses around 2010.
- In Dutch surveys of 2015, 2016 and 2017, the average KAP was, respectively, 1.12 Gy cm^2^ (11 hospitals), 1.26 Gy cm^2^ (8 hospitals) and 0.99 Gy cm^2^ (8 hospitals) [71–73]. As 50% or more of the hospitals had a KAP lower than 1.0 Gy cm^2^, KAP values lower than 1.5 Gy cm^2^ should be easily attainable. This value is currently the (still conservative) Dutch DRL target [74]. It was used in calculations for 2017, together with a high voltage of 80 kV, an anode angle of 16°, 3.5 mm Al total filtration and a 105-cm focus-detector distance.
- Averages from two rooms in our hospital gave values for 2018; the technique parameters are given in Table 2. The latter have essentially remained the same since 2011 to the apparent satisfaction of the radiological staff [7]. For general information on dose reduction in digital radiography by Cu-filtration, see, e.g. Martin [75] and Kawashima [76].

**Table 2:** Technique parameters AP pelvic radiography in our hospital (MUMC+)^a^

Room	*n*	kVp	Tube current	*t*_exposure_	Tube load	KAP	FID	KfiA	ESAK
			mA	ms	mA.s	Gy cm^2^	cm	mGy	mGy
1	238	81 ± 1	806 ± 15	20 ± 13	16 ± 10	0.48 ± 0.29	126 ± 11	0.52 ± 0.31	0.78 ± 0.46
2	110	85 ± 0	472 ± 2	49 ± 28	23 ± 13	0.70 ± 0.41	142 ± 7	0.61 ± 0.36	0.91 ± 0.54

^a^ Inherent filtration X-ray tube 3 mm Al, added filtration 0.1 mm Cu, anode angle 16°, a 25-cm distance from skin on X-ray entrance side to image receptor is assumed, backscatter factor 1.49

*KAP* kerma area product, *FID* ray focus to image receptor distance, *KfiA* kerma free in air at entrance position on skin (patient removed), *ESAK* entrance surface air kerma including backscatter (=KfiA × backscatter factor)

### Optimisation of AP pelvic radiography

**Fig. 2:**
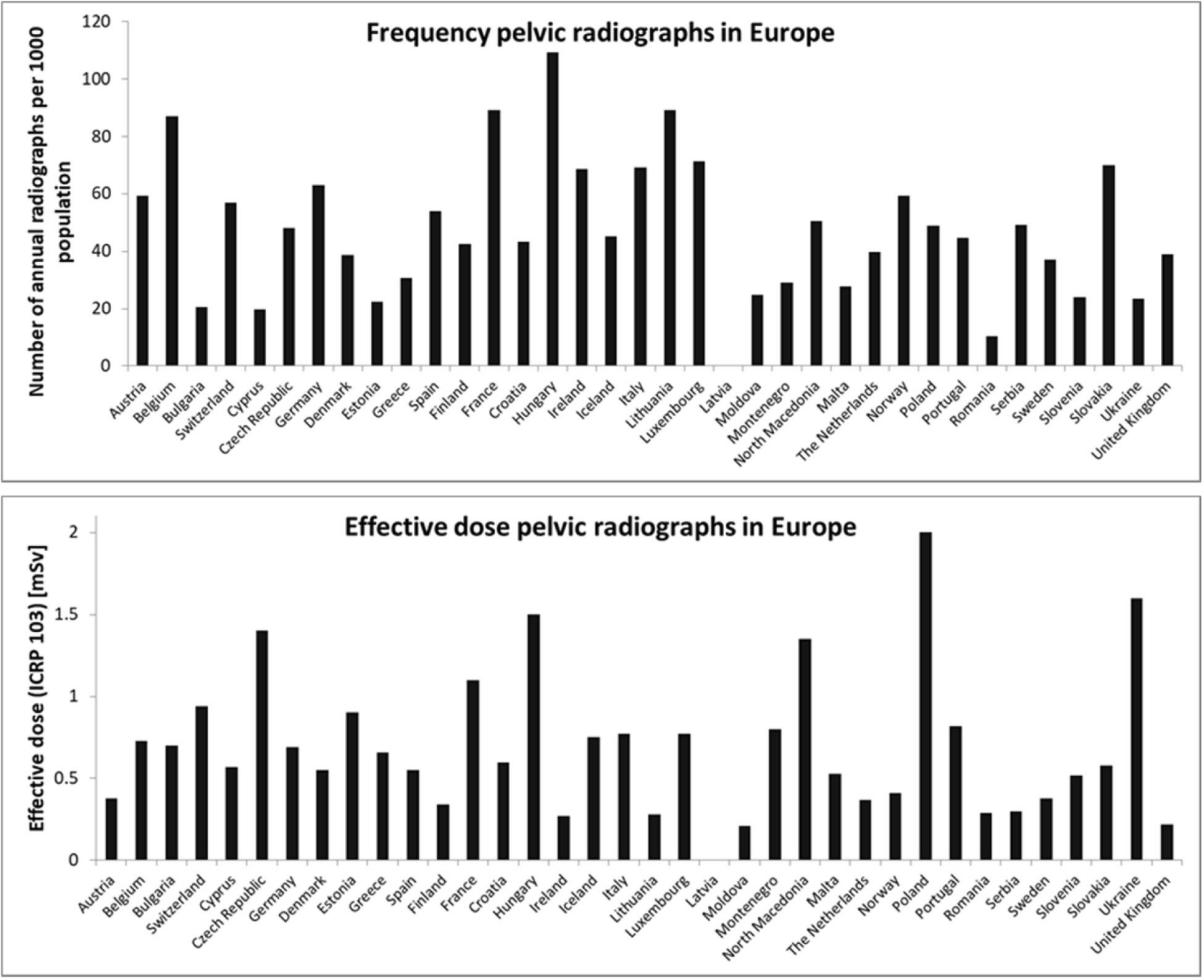
Annual frequency of pelvic radiographs per 1000 population in European countries (top). Effective dose of pelvic radiographs in European countries (bottom). According to RP180, data from 2007 to 2010 [77]

The potential for optimisation was assessed starting from European data presented in: “Medical Radiation Exposure of the European Population, Radiation Protection report No 180” [70, 77], hereafter referred to as RP180. The final documents are from 2015, reporting data collected in surveys during 2007–2010. Amongst other data, RP180 provides the annual frequency and effective dose of several X-ray examinations for 35 countries in Europe (573 million inhabitants; data for Latvia are missing). Figure 2 shows data for radiography of the pelvis.

The effective dose data in Fig. 2 (bottom), being from 2007 to 2010, will partly stem from screen-film systems, generally with a speed of about 400, and partly from digital systems often also set at speed 400. For skeletal (including pelvic) radiography, however, speed 800 with image quality (nearly) equalling speed 400 screen-film may have been used [78].

The potential of dose optimisation is illustrated by calculating the cumulative effective dose from all EU countries for four levels of optimisation. First, the RP180 data as reported [77] are used. In the second scenario, all European countries are supposed to expose pelvic radiographs at the level of the most common DRL in Europe, i.e. a KAP of 3.0 Gy cm^2^ [70]. In scenario 3, the exposure level equals the already discussed Dutch target DRL, i.e. a KAP of 1.5 Gy cm^2^, and in scenario four all exposure parameters are again taken from our hospital (Table 2). In all four scenarios, the national pelvic radiograph frequencies remained as reported in RP180.

In a fifth scenario, the radiograph frequency was harmonised by setting it for all countries to the Dutch value, while national effective doses as reported in RP180 were used. For justification of using Dutch references, see [79]. Looking at effective dose makes sense because gonad doses roughly scale with it: the absorbed dose of the testes varies between 8 and 14 times the effective dose, the ovary dose between 1 and 2.5 times as will be shown hereafter.

### Effect of gonad shielding on health risk

The motivation for reintroducing gonad shielding in the 1950s was reduction of hereditary risk. Risk caused by radiation is commonly assessed as a “detriment-adjusted risk”, which weighs not only life lost from fatal cancers and heritable effects, but also takes the reduced quality of life due to non-fatal cancers and heritable effects into account [80]. Around 2011, Frantzen et al. performed such a risk assessment for children [7]. Here it is done for adults and the exposure conditions described under “Radiation dose of an AP pelvic radiograph over time”.

In our calculations, 5.40 × 10^−3^ Sv^−1^ was used as the detriment-adjusted nominal risk coefficient for heritable disease [35]. This value holds for the reproductive population for which shielding is relevant. As risk for cancer the value for the whole population, 5.5 × 10^−2^ Sv^−1^, was taken [35]. Gonad shields were assumed to have the (optimal) protection factors of 0.95 for the testes and 0.5 for the ovaries [59].

## Results

### Radiation dose of an AP pelvic radiograph over time

**Fig. 3:**
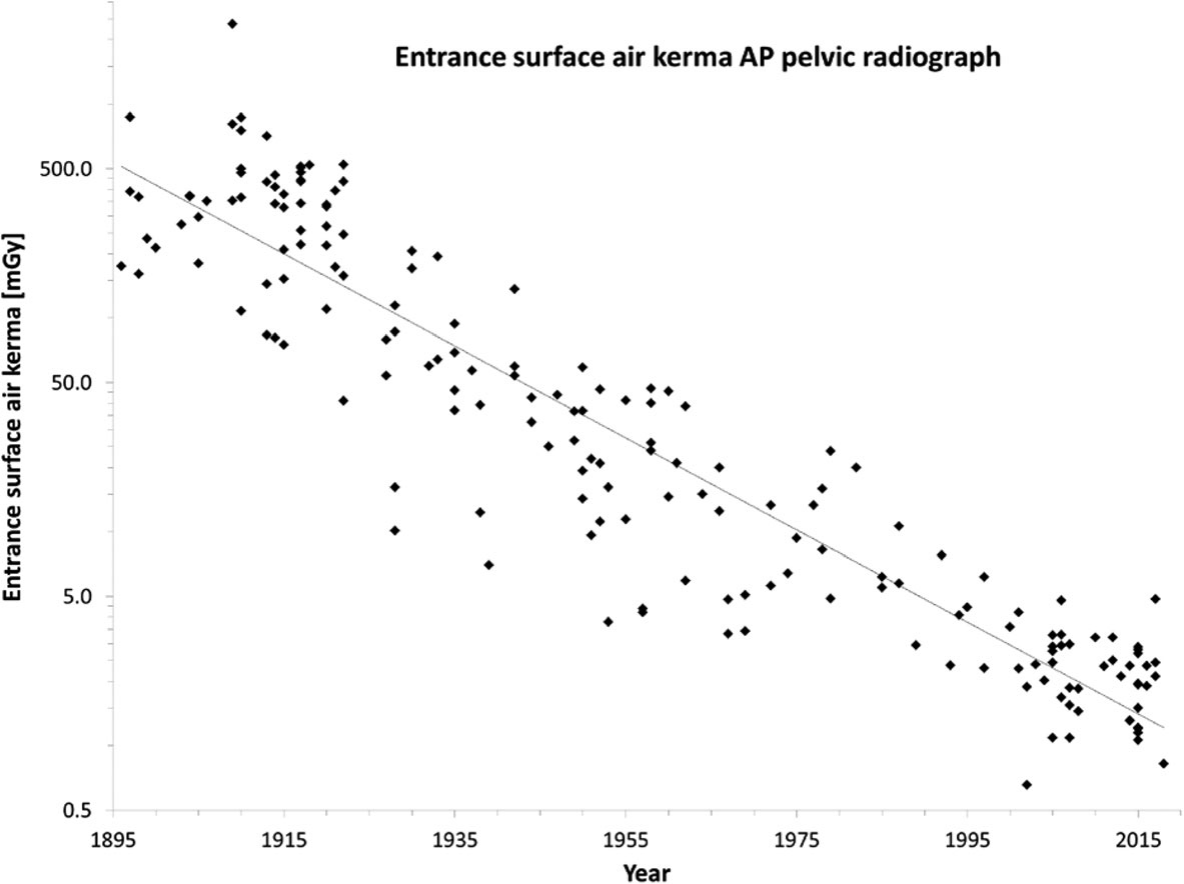
Entrance surface air kerma including backscatter of an AP pelvic radiograph over the years (*n* = 182). Please note the logarithmic *y*-axis. The solid line is a fit of a simple exponential function to all data (“exponential regression”) [61]

Figure 3 shows all dose data, reconstructed and retrieved, as “entrance surface air kerma including backscatter” (ESAK) [61]. An enormous spread in dose can be observed at all times and an average dose decrease between 1896 and 2018 by a factor of about 400.

Table 3 shows doses over time, with at its bottom the dose reduction that has been achieved since 1905. Note that the relative reduction in ESAK is different from that in KAP due to differences in backscatter factor and focus-skin distance.

**Table 3:** Mean dose data AP pelvic radiograph in absence of gonad shielding

Year	Source of data	ESAK (mGy)	KAP (Gy cm^2^)	Effective dose (ICRP 103) (mSv)	Absorbed dose	
					Testes^a^ (mGy)	Ovaries^a^ (mGy)
1905	Beck, Biddle, Albers-Sch.^b^	341	173	11.4	149	13
1958	Janker, Lincoln^b^	25	15.4	1.32	15	2.1
2010	“European” DRL^c^	5.4	3.0	0.52	4.5	1.2
2017	Dutch target DRL^d^	2.7	1.5	0.26	2.3	0.61
2018	MUMC+	0.82	0.55	0.095	0.74	0.24
	Dose 2018/Dose1905	0.22%	0.26%	0.86%	0.48%	2.0%

*ESAK* entrance surface air kerma which includes backscatter (dose in air but on the skin) [61], *KAP* product of kerma free in air and area of primary X-ray beam, *DRL* diagnostic reference level

^a^With optimal shielding, these doses might be reduced by about 95% and 50%, respectively

^b^Pulsed voltages were used. The equivalent DC voltage was calculated on the basis of effective dose in the same way as kV peak was converted to DC-kV on the basis of kerma free in air [61]

^c^Most common DRL in Europe (KAP = 3.0 Gy cm^2^) [70]

^d^Conservative Dutch target DRL (KAP = 1.5 Gy cm^2^) [74]

### Optimisation of AP pelvic radiography

Table 4 shows collective effective doses in Europe for different degrees of optimisation. Note that “optimisation” may include the installation of a modern high-power, digital system. Scenario 4 illustrates that such a modern system, properly optimised, can lower the European collective effective dose by a factor of nine compared to the value calculated using RP180 data from 2007 to 2010. Scenario 5 in Table 4 shows that harmonising the frequency of pelvic radiographs in all European countries to the Dutch value of 39.8 per 1000 persons, results in a dose reduction of nearly 30%. The average frequency in Europe was 54.3 per 1000 persons.

**Table 4:** Cumulative effective dose caused by AP pelvic radiography in 35 European countries

Scenario	Source effective dose per radiograph	Source of annual frequency of pelvic radiographs (RP180)	Cumulative effective dose^a^, kmanSv	Percentage
1	Individual countries (RP180)^b^	Individual countries	26.4	≡100
2	“European” DRL (RP180)^c^	,,	16.1	61
3	Dutch target DRL^c^	,,	8.0	30
4	MUMC+	,,	3.0	11
5	Individual countries (RP180)	The Netherlands (RP180)^d^	18.7	71

^a^ Cumulative effective dose is the sum of the effective dose over all exposed persons in the 35 countries (k in kman-Sv stands for kilo, i.e. 1000)

^b^ Assuming AP projection dominates pelvic effective dose given in RP180

^c^ Most common DRL in Europe is a KAP of 3.0 Gy cm^2^, also in the Netherlands [70]. The Dutch target is 1.5 Gy cm^2^, however [74]

^d^ The annual frequency of pelvic radiographs in the Netherlands is 39.8 per 1000 population [77]

### Effect of gonad shielding on health risk

Table 5 shows the strong decrease in detriment-adjusted risk resulting from technological developments and optimisation. Today, even assuming optimal shielding and no negative side-effects as done for Table 5, gonad shielding causes a very small reduction in risk only.

**Table 5:** Detriment-adjusted risks for adults of reproductive age caused by AP pelvic radiography

Year	Origin of data at basis of calculation	Total risk X-ray without shielding^a^		Total risk X-ray with shielding^b^		Reduction risk by shielding gonads^c^	
		Males	Females	Males	Females	Males	Females
		per 10^6^	per 10^6^	per 10^6^	per 10^6^	per 10^6^	per 10^6^
1905	Beck, Biddle, Albers-Sch	1075	341	308	307	767	35
1958	Janker, Lincoln	116	47	39	41	77	5.6
2010	“European” DRL^d^ (RP180)	40	23	17	19	23	3.3
2017	Dutch target DRL^d^	20	11	8.5	9.7	12	1.7
2018	MUMC+	8.6	5.4	4.0	4.6	4.6	0.77

^a^ Using abbreviations *R* = detriment-adjusted risk, *E* = effective dose and *H* = equivalent dose, the risk was approximated as *R*_male_ = 5.5 × 10^−2^ × {*E* − 0.04 × (*H*_Testes_ + *H*_Ovaries_)} + 5.4 × 10^−3^ × *H*_testes_ and analogously for females. Applied for instance to “1958 males without shielding”, this gives *R*_male_ = 5.5 × 10^−2^ × {1.32 × 10^−3^ − 0.04 × (15 × 10^−3^ + 2.1 × 10^−3^)} + 5.4 × 10^−3^ × 15 × 10^−3^ = 116 × 10^−6^. Note that we used data from Table 3 and that the equivalent dose equals the absorbed dose multiplied by the relative biological effectiveness of the radiation causing the absorbed dose. For X-rays, this factor is 1 Sv/Gy, so absorbed and equivalent dose are numerically equal

^b^ With gonad shielding, the last term in *R*_male_ is modified into: 5.4 × 10^−3^ × (1 − GS) × *H*_Testes_, with GS the shielding factor of 0.95 for males. Analogously for females, but with GS = 0.5

^c^ Decrease in total risk resulting from the reduction in hereditary (“gonad”) risk by shielding

^d^ Most common European DRL, i.e. KAP = 3.0 Gy cm^2^; Dutch target DRL is KAP = 1.5 Gy m^2^

## Discussion

When AP pelvic radiography is performed with modern and optimised X-ray systems, the reduction in hereditary risk by gonad shielding in women is so small that shielding can safely be discontinued. For men, the risk reduction can be larger but is still so small that it is doubtful whether the benefits outweigh the potential negative side-effects of using a shield. Several factors led to this state of affairs.

First, technological developments enabled an enormous reduction in the dose needed for a pelvic radiograph, as illustrated in Fig. 3 and Table 3. All dose cutbacks either directly resulted from these advances (e.g. higher sensitivity of image receptors and digital image processing) or were facilitated by them (e.g. higher power allowed increase of focus-patient distance and more filtration).

Second, optimisation lowered doses still further as shown by numerous studies as well as Table 4. Unfortunately, this potential has never adequately been exploited as illustrated by the large spread in Fig. 3 (at all times!) and Fig. 2 (around 2010). This is something the radiological profession should take to heart given longstanding guidance and legislation. The “As Low As Reasonably Achievable” (ALARA) principle goes back to 1966 [81], the requirement to optimise is from 1973 [82]. The large variability in frequency of pelvic radiographs reported in EU report RP180 for comparable EU countries is also unsatisfactory (Fig. 2, top), especially because 17 countries answered the question “Does the reimbursement system affect the frequency of examinations?” with “yes”. This seems to imply that earnings affect study justification. An identical frequency of pelvic radiographs throughout the EU, equal to the Dutch value, could already lower the collective effective dose by 29%.

Third, more recent insights into radiation biology have led to lower estimates of hereditary risks. According to current understanding, radiation-induced mutations generally do not come to expression in descendants, because, in the words of ICRP 103: “Most radiation-induced mutations are large multigene deletions, which are more likely to cause multisystem developmental abnormalities rather than single-gene (i.e., Mendelian) diseases. Importantly, only a fraction of these are likely to be compatible with live births.”, and “Nearly all chronic diseases have a genetic component, but because most of these are multigenic and multifactorial, the mutation component (i.e., the responsiveness of these diseases to an alteration in mutation rate) is small, so that chronic diseases respond only minimally to a radiation-induced increase in mutation rate” [35].

The effectiveness of diagnostic reference levels (DRLs) in optimisation may need a closer look. DRLs were introduced by the ICRP in 1990 [38] and further addressed in 1996 [83], 2001 [84] and 2017 [85]. The Euratom Council Directive from 2013 [86] reiterated the importance of DRLs, and the European Society of Radiology (ESR) tried (and tries) to help their implementation in radiological practice with their programme “Eurosafe Imaging” [87]. Success can be claimed to the extent that DRLs were applied in about 80 to 90% of the institutions surveyed by the ESR in 2019 [88]. However, by its approach, i.e. setting the 75 percentile of the dose distribution at some time as the DRL and then “correcting” the systems with doses higher than the DRL—after which the whole cycle should be repeated—the average dose decreases only very slowly. A recent (2019) and large study by Schegerer et al. [89] may be seen as illustrative: nearly 30 years after the introduction of the DRL, the ratio of the 25th and 75th percentiles for “pelvis AP/PA” (and most other conventional X-ray projections) still exceeded a factor 2, signalling a broad distribution of doses (in line with Fig. 3). In conclusion, local optimisation, which for instance resulted in the technique parameters of Table 2, is probably much more efficient than following the DRL approach. The strength of DRLs is eliminating bad practices.

The question remains how to proceed with gonad shielding. Several studies pointed to inadequate placement of gonad shields, the concomitant loss of diagnostic information and the low doses currently involved, but were cautious with their advice. A few suggest reconsidering or ending the practice in female children [6–9, 11–13], in male children [7, 9, 12] or in all [3, 10]. Marsh and Silosky are more outspoken when referring to the officially still endorsed practice of gonad shielding as “…the folly of its continued use…” [4]. They question the linear-no-threshold model and the cumulative nature of small doses, maybe rightly, but in our opinion, it is wise to abide by the prevailing views disseminated by the international organisations in the radiation protection field. Marsh and Silosky further argue that the benefits are small or non-existent and that shielding involves considerable risks. Risks certainly exist, but unfortunately they are very difficult to assess in a quantitative way. Reported negative effects (“risks”) of shielding include [7] the following: testes dose reduction of less than 95% due to misplaced shields (e.g. 77% in 10–15-year olds) [7], the need of retakes (Gürsu et al. reported a retake rate of 3% in children up to 17 years) [90], a dose increase if the shield covers (part of) the automatic exposure control (AEC) detector [13], and loss of diagnostic information, and distraction of the technologist by handling the shield. The small risk reductions seen at the bottom of Table 5 (a risk < 1 × 10^−6^ is considered inconsequential [91]) have to be viewed in the light of such effects. Note also that these reductions are only obtained under ideal shielding circumstances. For females, the conclusion is straightforward, but for males it is less obvious. Given the information above, and that the AEC detector generally is not behind the (shielded) testes, it is hardly to be expected that poor positioning, retakes and AEC coverage would decrease the (average) male shielding-factor from 0.95 to below 0.5. The benefit of shielding shown in Table 5 might then actually be up to about 50% lower. But other, probably rare effects caused by information loss and user distraction may be more important. Two hypothetical examples might give an idea of what could go wrong due to using a gonad shield. A seldom, but not impossible, fatal accident could be the missing of a Ewing sarcoma in the pubic bone of a boy with pain in his groin because the shield covered the lesion. Or an infant, or an unconscious person, could tumble from the table because the radiographer was picking up a shield lying outside his reach before some fixation of the patient was arranged. But how often will such or other serious incidents happen? Or how often, and how seriously, will shielding hamper diagnostic evaluation? One does not know, and although this is clearly a limitation of the study, it is evident that harm is possible and that already very few incidents per million radiographs would undo the small benefit calculated in Table 5.

After discontinuing gonad shielding, as our hospital already did in 2011, patients (or their parents) may perceive not using a shield a serious neglect. They must therefore be informed and possibly reassured, for instance by giving examples of effective doses of a similar magnitude received during common activities (see, e.g. [7], Table 7). In our experience, not shielding quickly becomes the new standard.

## Conclusions

Modern equipment and optimisation are keys to reducing radiation risk in pelvic radiography. When their full potential is exploited, the decrease in detriment-adjusted risk achievable by gonad shielding is so small that, in the light of negative side-effects, ending the practice seems justifiable.

## Data Availability

References are given for all data, except for the dose data on pelvic radiography originating from our own hospital. The latter are available from the first author.

## Abbreviations

AAPM: American Association of Physicists in Medicine
AEC: Automatic exposure control
ALARA: As Low as Reasonably Achievable
AP: Anteroposterior
DRL: Diagnostic reference level
E: Effective dose
ESAK: Entrance surface air kerma (dose in air at the skin, including backscatter)
EU: European Union
FID: X-ray focus to image receptor distance
GSD: Genetically significant dose
H_E_: Effective dose equivalent
IAEA: International Atomic Energy Agency
ICRP: International Commission on Radiological Protection
KAP: Product of kerma free in air and X-ray beam area
KfiA: Kerma free in air
MUMC+: Maastricht University Medical Center
PCXMC: A Monte Carlo programme for calculating patient doses in medical x-ray examinations
PMMA: Polymethylmethacrylate (Acrylic, Lucite, Perspex)
R: Detriment-adjusted risk
RP180: Medical Radiation Exposure of the European Population, EU Report 180 (2015)
UNSCEAR: United Nations Scientific Committee on the Effects of Atomic Radiation
